# Brown fat fuels the fire in fever

**DOI:** 10.1016/j.jlr.2024.100658

**Published:** 2024-09-26

**Authors:** Samantha J. Krysa, Jonathan R. Brestoff

**Affiliations:** Department of Pathology and Immunology, Washington University School of Medicine, St. Louis, MO, USA

**Keywords:** Brown adipose tissue, BAT, fatty acid oxidation, fever, febrile, immunometabolism

## Abstract

Fever is a host-pathogen defense mechanism in which the immune system drives a physiologic increase in core body temperature. For over 50 years, it has been known that the temperature of brown adipose tissue (BAT) is increased during the febrile response. However, recent studies suggested that the primary thermogenic protein Uncoupling protein 1 in brown adipocytes does not contribute to fever induction in mice, casting doubt about the functional contribution of BAT to fever. In a new set of studies, Li *et al.* (2024) provide compelling evidence that fatty acid oxidation is markedly increased in BAT in a *Salmonella* infection model of fever and strongly suggest that metabolic adaptation in BAT may play a critical role in the febrile response. This article re-opens the debate about how thermogenic and metabolic programs in BAT contribute to fever and raises new questions about whether BAT contributes to host defense against pathogens.

Fever is a physiologic elevation in core body temperature and is a hallmark characteristic of the host response to infections (1). The induction of fever is believed to be coordinated by immune cells that release pyrogenic cytokines, such as tumor necrosis factor-α, interferon-γ, interleukin (IL)-1β, and IL-6, which act upon the central nervous system to increase core body temperature (2). Given the critical nature of core body temperature regulation in mammalian physiology and survival, an intricate set of mechanisms have evolved to induce fever, maintain a higher core body temperature, and restore core body temperature to normal levels after the resolution of the infection and associated immune responses (1).

Several years ago, a series of studies addressed the hypothesis that the thermogenic organs such as brown adipose tissue (BAT) and its primary thermogenic protein Uncoupling protein 1 (UCP1) contribute to the induction of fever. A role for BAT in fever was suggested over 50 years ago by Székely *et al.* (1973) in studies showing that increased BAT temperature precedes the increase in core body temperature during fever induction (3). It was also shown that lipopolysaccharide (LPS) induces fever in guinea pigs and rats, a response that was blunted with β-blockers that inhibit sympathetic nervous system (SNS)-mediated activation of brown adipocytes (4). However, more recently, it was shown that UCP1-deficient mice have similar core body temperatures as wildtype controls after injection with IL-1β or LPS (5, 6, 7). These latter studies suggested that BAT and UCP1 do not contribute to the febrile response in mice, and research on the functional role of BAT in contributing to fever has since waned.

In this issue of *Journal of Lipid Research*, Li *et al.* re-opened this subject and shed new light on the involvement of BAT in fever, following infection with the gram-negative bacterium *Salmonella enterica* serovar Typhimurium (S.tm) (8). This study used a transgenic mouse model of human-like lipoprotein metabolism (APOE∗3-Leiden.CETP) and demonstrated that S.tm infection increases fatty acid oxidation in BAT while reducing circulating triglycerides (TG), a finding they attributed to the highly elevated TG uptake and fatty acid oxidation by brown adipocytes. They also found that BAT activity and uptake of TG-derived fatty acids was positively correlated with core body temperature during infection-induced fever. Furthermore, increased TG-derived fatty acid uptake was not associated with increased BAT mass, suggesting that the tissue is oxidizing these fatty acids for heat production. S.tm infection led to increased SNS activation in BAT as evidenced by increased expression of tyrosine hydroxylase, increased activation of factors downstream of β_3_ adrenergic receptor signaling (e.g., phosphorylation of cAMP response element-binding protein), a gene expression signature associated with increased sympathetic tone, and increased expression of UCP1 in BAT during infection-induced fever. This constellation of findings supports the idea that S.tm. infection is associated with increased SNS-mediated activation of BAT, which may oxidize circulating lipids to contribute to the thermogenic response during infection (Fig. 1).

**Fig. 1:**
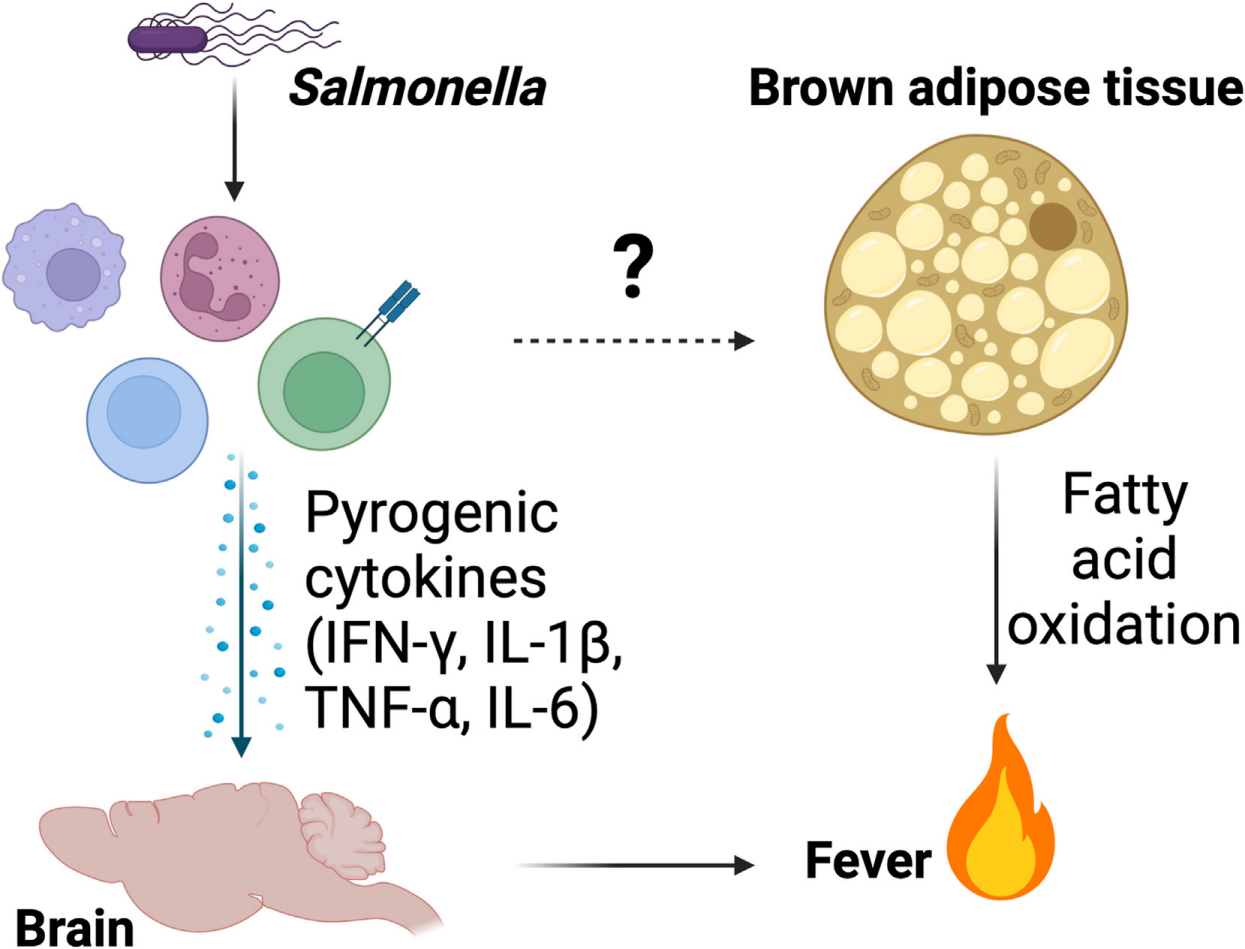
Brown adipose tissue oxidizes lipids to contribute to fever. The immune system initiates a fever by releasing pyrogenic cytokines, such as tumor necrosis factor (TNF)-α, interferon (IFN)-γ, interleukin (IL)-1β, and IL-6. The fever response is driven by the central nervous system, which also controls brown adipose tissue (BAT) activation. Brown adipocytes oxidize lipids in the setting of fever, which may help to sustain the elevation in core body temperature. It remains unknown whether the immune system coordinates the metabolic adaptation in BAT to support the febrile response. S.tm, *Salmonella enterica* serovar. Created with BioRender.com.

Taken together, these data highlight a potential role for BAT thermogenesis in the febrile response following infection with S.tm. They also raise interesting new questions that should be addressed in future studies. Previous studies indicated that UCP1 is not required for the induction of febrile responses 1–8 h after administration of recombinant IL-1β or LPS (5, 6, 7), suggesting that UCP1 is not required for the initial phases of induction of fever in these noninfectious models of fever. Do BAT and UCP1-dependent thermogenesis contribute to the induction of fever in infection models, such as S.tm? Does this thermogenic pathway contribute to the later phases of fever to maintain elevated core body temperature? Is brown fat activation increased in people in the setting of fever?

Recent studies also have revealed that UCP1 is not the only thermogenic protein in beige and brown adipocytes. Deletion of UCP1 leads to compensatory increases in the expression of creatine kinase B (CKB) and a CKB-mediated futile cycle (9, 10). This CKB thermogenic mechanism allows UCP1-deficient animals to partially defend their core body temperature and survive in cold environmental temperatures, but deletion of both UCP1 and CKB in adipocytes leads to rapid hypothermia and death in the cold (11). It is possible that this CKB futile cycle compensated for the loss of UCP1 in prior studies reporting that UCP1 is dispensable for the induction of IL-1β- or LPS-induced febrile responses. What are the differential contributions of UCP1 and CKB in BAT in regulating the induction and/or maintenance of fever? Do these thermogenic mechanisms contribute to fatty acid oxidation in BAT in fever?

Fever is considered a host-defense mechanism against pathogens by accelerating the immune response and confers a survival benefit during infection (1). As fever is initiated by the immune system, an exciting future direction will be to determine whether an immunologic mechanism drives the increase in BAT fatty acid oxidation in the context of fever. Macrophages in BAT are known to regulate sympathetic tone by degrading SNS-derived catecholamines, and this response regulates rates of lipolysis in brown adipocytes (12, 13). Future studies should determine whether macrophages or other immune cells in BAT promote fatty acid oxidation and increase core body temperature in febrile settings. It seems logical that the immune system could not only trigger a fever but also coordinate the metabolic adaption required to maintain elevated core body temperature, and this topic remains to be explored.

## Conflicts of interest

J. R. B. has pending patent applications related to the treatment of obesity (63/625,555) and allergic diseases (US20210128689A1), is a consultant for Columbus Instruments, has been a consultant for DeciBio in the past 12 months, is a member of the Scientific Advisory Board for LUCA Science Inc., receives research support from LUCA Science Inc. and Edgewise Therapeutics Inc for projects unrelated to this manuscript, receives royalties from Springer Nature Group, and is an inventor of technology licensed to Columbus Instruments with royalty rights. S. J. K. declares that they have no conflicts of interest with the contents of this article.
